# Identification of Zoonotic Genotypes of *Giardia duodenalis*

**DOI:** 10.1371/journal.pntd.0000558

**Published:** 2009-12-01

**Authors:** Hein Sprong, Simone M. Cacciò, Joke W. B. van der Giessen, on behalf of the ZOOPNET network and partners

**Affiliations:** 1 Laboratory for Zoonoses and Environmental Microbiology, National Institute for Public Health and Environment (RIVM), Bilthoven, The Netherlands; 2 Department of Infectious, Parasitic and Immunomediated Diseases, Istituto Superiore di Sanità, Rome, Italy; Faculté de Médecine, Université de la Méditerranée, France

## Abstract

*Giardia duodenalis*, originally regarded as a commensal organism, is the etiologic agent of giardiasis, a gastrointestinal disease of humans and animals. Giardiasis causes major public and veterinary health concerns worldwide. Transmission is either direct, through the faecal-oral route, or indirect, through ingestion of contaminated water or food. Genetic characterization of *G. duodenalis* isolates has revealed the existence of seven groups (assemblages A to G) which differ in their host distribution. Assemblages A and B are found in humans and in many other mammals, but the role of animals in the epidemiology of human infection is still unclear, despite the fact that the zoonotic potential of *Giardia* was recognised by the WHO some 30 years ago. Here, we performed an extensive genetic characterization of 978 human and 1440 animal isolates, which together comprise 3886 sequences from 4 genetic loci. The data were assembled into a molecular epidemiological database developed by a European network of public and veterinary health Institutions. Genotyping was performed at different levels of resolution (single and multiple loci on the same dataset). The zoonotic potential of both assemblages A and B is evident when studied at the level of assemblages, sub-assemblages, and even at each single locus. However, when genotypes are defined using a multi-locus sequence typing scheme, only 2 multi-locus genotypes (MLG) of assemblage A and none of assemblage B appear to have a zoonotic potential. Surprisingly, mixtures of genotypes in individual isolates were repeatedly observed. Possible explanations are the uptake of genetically different *Giardia* cysts by a host, or subsequent infection of an already infected host, likely without overt symptoms, with a different *Giardia* species, which may cause disease. Other explanations for mixed genotypes, particularly for assemblage B, are substantial allelic sequence heterogeneity and/or genetic recombination. Although the zoonotic potential of *G. duodenalis* is evident, evidence on the contribution and frequency is (still) lacking. This newly developed molecular database has the potential to tackle intricate epidemiological questions concerning protozoan diseases.

## Author Summary

*Giardia duodenalis* is a parasite causing a gastrointestinal disease in humans, pets, livestock, and wildlife. The role of animals in human disease is unclear, because *Giardia* from humans and animals is morphologically indistinguishable. An international consortium of both veterinary and public health institutions built a web-based database, where molecular and epidemiological data are combined. After extensive genetic characterization, the zoonotic potential of *Giardia* became evident, but data on frequency and role in epidemiology is (still) lacking. Surprisingly, mixtures of *Giardia* genotypes in individual hosts were frequently observed, and have important implications for the etiology of *Giardiasis*. Possible explanations are the uptake of mixtures of *Giardia* genotypes by one host, or subsequent infection of an already infected host, likely without overt symptoms, with a different *Giardia* species, which may cause disease. We demonstrated that collaborative, human and veterinary health integrated databases have the potential to tackle intricate epidemiological questions concerning parasitic diseases, as was demonstrated for *G. duodenalis* in the present study.

## Introduction

*Giardia* is a genus of intestinal flagellates that infect a wide range of vertebrate hosts. The genus consists of six species, which are distinguished on the basis of the morphology and ultra-structure of their trophozoites [1]. *Giardia duodenalis* (syn. *G. intestinalis*, *G. lamblia*) is the only species found in humans, although it exhibits a wide host range being found in many other mammals. *G. duodenalis* is the etiological agent of giardiasis, a gastrointestinal infection in humans ranging from asymptomatic to severe diarrhea as well as chronic disease [2]. Giardiasis represents a major public health concern in both developing and developed countries [3],[4]. The economic losses, both direct and indirect, caused by this widespread parasitic infection are considerable. Children are at most risk from the clinical consequences of *G. duodenalis* infection, particularly those in developing countries and living in disadvantaged community settings [5]. In population- and general practitioner-based studies in The Netherlands, *G. duodenalis* was identified as the most important gastrointestinal parasitic pathogen [6],[7]. Paradoxically, the diagnosis of giardiasis is not routinely carried out, due to lack of awareness and the similarity of symptoms with other gastro-enteritis diseases. *G. duodenalis* is also of significant clinical and economic importance in livestock and pet animals [8]–[10].

*Giardia* has a simple life cycle comprising rapidly multiplying, non-invasive trophozoites on the mucosal surface of the small intestine, and the production of environmentally resistant cysts that are passed with the host faeces. Infectious cysts are transmitted by the faecal-oral route, either by direct contact or by ingestion of contaminated food or water [11]. Illness from this parasite arises through infection in two broad settings: outbreaks and (sporadic) endemic transmissions. Outbreaks are most frequently waterborne and caused by contamination of drinking water, although other transmission routes have been implicated as well [1],[12],[13]. One complicating factor is that the number of asymptomatic carriers, and their role in the spread of the infections, are not clear [6],[7],[12],[14].

*G. duodenalis* can be considered as a species complex, whose members show little variation in their morphology, yet can be assigned to seven distinct assemblages (A to G) based on genetic analysis [15]. Assemblages A and B are responsible for human infection, and are also found in a wide range of mammals. The remaining assemblages show more restricted host ranges: C and D are found in canids, E in livestock, F in cats, and G in rodents [16]. Genetic characterization has been extensively used to assess the role of animals in the epidemiology of human infection and to develop tools for tracing sources of infection. However, the zoonotic potential of *G. duodenalis* is still under debate, particularly the role of domestic animals. Transmission may occur from animals to humans or from humans to animals. Alternatively, humans and animals may be infected with host-adapted genotypes only. For example, transmission of *Giardia* from beavers to humans via drinking water was postulated [17],[18]. In endemic areas where humans and animals live closely together, transmission from human to animals or vice versa may occur [19],[20]. Also, the existence of host-adapted *Giardia* genotypes has been reported [21],[22]. Until now, the majority of molecular epidemiological studies have been based on the analysis of a single marker from a limited number of isolates. Furthermore, the genetic variability and the usefulness of the different loci in identifying genotypes have not been systematically evaluated. Finally, it remains unclear to what extent allelic sequence heterozygosity (ASH) and genetic exchanges contribute to the genetic variation found in *Giardia* [23]. In this study the zoonotic potential of *G. duodenalis* is investigated at different levels of resolution (single and multiple loci on the same dataset). Zoonotic potential is defined here as a *G. duodenalis* genotype, which has been isolated from both human and animal, sources, and doesn't take into account other epidemiological parameters (such as time and geographical origin).

A European network of public and veterinary health Institutions from 9 European countries that focuses on zoonotic protozoan parasites (the ZOOnotic Protozoa NETwork, ZOOPNET) has been established (Sprong et al. submitted) as part of MedVetNet, a European network of excellence working for the prevention and control of zoonoses and food borne diseases. The aims of ZOOPNET were (i) to harmonize the methodology for the detection and control of *Giardia* and *Cryptosporidium*, (ii) to investigate the molecular epidemiology of these infections, and (iii) to study the role of animal sources in human disease. A molecular epidemiological database was built in the course of the project, currently containing information on 2476 *Giardia* isolates, which encompass 3886 sequences, and on 1024 *Cryptosporidium* isolates, for a total of 1664 sequences. The ZOOPNET-database differs from a representative (e.g. Genbank) or a genomic (e.g. GiardaDB) database [24], as it aims to collect epidemiological data linked to a few molecular markers from as many field isolates as possible. A field isolate can be described best as a DNA sample isolated from a human, animal or environmental source. This implies that an isolate may contain more than one *G. duodenalis* species or genotypes. A part of the database is already publicly available (https://hypocrates.rivm.nl/bnwww/MedVetNet/). Currently, a more user-friendly web-based database which not only contains all the molecular epidemiological data used in this study, but also allows public and veterinary health researchers to BLAST their sequences in the database, to perform basis phylogenetic analysis and to submit their own data into the database.

In the present study, the genetic diversity and geographic distribution of *G. duodenalis* of human and animal origin, and the potential for zoonotic transmission, were assessed by different molecular genotyping methods.

## Methods

### Origin of the isolates

Giardia isolates of human and animal origin were collected by Public and Veterinary Health Institutions from the European countries represented in the network, as well as and from external research groups on a voluntary basis. Epidemiologic and molecular data were submitted using an Excel-based file, and form the basis of the information present in the database (Sequences and data used for this study are available on request). Furthermore, *Giardia* sequences were retrieved from the Genbank database. A selection of these sequences was made using the same strategy as previously described [25]. For example, sequences that were too short to cover regions of variation within any given assemblage were used only for analysis at the level of that assemblage, but not at the level of sub-assemblage. In addition, when multiple, identical sequences from any given isolate were deposited in Genbank, only the longest available sequence was retrieved. Although Genbank sequences constitute ∼45% of the database, limited epidemiological data (mainly country and source of isolation) are available for those isolates. All molecular epidemiological data were stored and analysed in Bionumerics (Version 5.10; Applied Math, Belgium). The contents of the database (February 2009) are described in the supporting information (Text S1).

### Sequence analysis

All of the *G. duodenalis* sequences were derived from genomic DNA. Most of the sequences were obtained from direct sequencing (occasionally cloned) of PCR products amplified from faecal samples. The sequences of reference isolates originated from laboratory strains, which were grown previously in culture or passaged through suckling mice. Each isolate was characterized using one to four of the most commonly employed genetic markers, which corresponds to portions of the small subunit ribosomal DNA (SSU-rDNA), beta-giardin (BG), glutamate dehydrogenase (GDH), and triose phosphate isomerase (TPI) genes [25]. All sequences were sorted into their different genes, assemblages, and sub-assemblages as well as alignments along the gene using previously defined references (Text S1). All of these markers, with the exception of the SSU-rDNA, have a high, though variable degree of genetic polymorphism [25], and were used to define sub-assemblages and subtypes. Sequences that were too short, or that contain ambiguous nucleotides which prevent their assignment to specific assemblage were excluded from further analysis [25]. Subtyping at the GDH locus was complicated by the use of different primers that amplify different portions of the gene, with only a partial overlap. In order to minimize these transitivity dilemmas, cluster analysis for each locus was performed using Unweigthed Pair Group Method with Arithmetic mean (UPGMA) and “most identical matches” as first and secondary criterion, respectively. A secondary criterion will be applied if two equivalent solutions will emerge from the first criterion.

The four markers used in this study are unlinked in the *G. duodenalis* genome, at least in the genome of assemblage A [22], which is a prerequisite for a multi-locus sequence typing scheme. The following *G. duodenalis* isolates were used as references for multi-locus sequence typing: for assemblage A, sub-assemblage AI, the axenic strains WB, Portland 1 and Ad-1 [26],[27]; for assemblage A, sub-assemblage AII, the axenic strains Bris-162, Bris-136 and KC8 [28]; for assemblage A, sub-assemblage AIII the isolate ISSGdA614 [22]; for assemblage B, sub-assemblage BIII, the strains BAH12 and Ld18 [26],[29]; and for assemblage B, sub-assemblage BIV, the strains Ad28 and Nij5 [26],[29].

## Results

The zoonotic potential of *G. duodenalis* can be inferred by comparing the genotypes of human and animal isolates. Here, genotyping was performed at different levels of resolution. First, for each marker all sequences were assigned to specific *G. duodenalis* assemblages (A to G) by comparison with previously defined sequences of reference strains [15]. Second, sequences of assemblages A and B were assigned to sub-assemblages AI, AII, AIII, BIII, and BIV using single-nucleotide polymorphisms (SNPs) from reference strains which were identified previously [22]. Third, since considerable sequence heterogeneity was also found within each sub-assemblage, subtypes (AS001, AS002, AS003, etc) were assigned to groups of sequences, on the basis of their similarity [22],[30],[31]. Fourth, genotyping data from different loci were combined to perform a multi-locus analysis.

### Typing at the assemblage level

The sequences from each of the four markers obtained from 2476 *Giardia* isolates were assigned to *G. duodenalis* assemblages A to G by comparison with previously defined reference strains (Text S1). The distribution of the assemblages within each source (corresponds to host or host group) was determined (Table 1). In humans (n = 1658), assemblage A (43%), B (56%) and to a much lesser extent C (0,1%), D (0,2%), E(0,2%), and F (0,2%) were found [32],[33]. All of these assemblages were also found in animals. Thus, at this very low level of resolution assemblages A to F can be considered zoonotic. The relative host range of a specific assemblage is calculated as the distribution of the sources within each assemblage (Table 2). The presented calculation does not take the absolute numbers of the sources in a population (e.g. number of cats compared to the number of humans in Europe) and the prevalence of giardiasis of each source into account. Still, assemblages C and D were mainly found in dogs (Table 2), assemblage E in livestock, F in cats and G in rodents (beavers and rats). These results are in agreement with previous findings [11],[16]. Remarkably, the host distribution of assemblage B is predominantly human and to a much lesser extent wildlife and dog (Table 2).

**Table 1:** Distribution of assemblages as percentage within each source.

Source	Cat	Cattle	Dog	Goat & Sheep	Human	Pig	Water	Wildlife	Other
A	**43**	**23**	23	**17**	**43**	**21**	**70**	**54**	**32**
B	2	2	9	1	**56**	0,7	**30**	**20**	**62**
C	3	0	**32**	0	0,1	0	0	2	0
D	2	0	**36**	0	*0,2*	0,7	0	2	0
E	1	**75**	1	**82**	*0,2*	**78**	0	**6**	**5**
F	**49**	0	0	0	*0,2*	0	0	0	0
G	0	0	0	0	0	0	0	16	0
*Total (n)*	*158*	*562*	*600*	*207*	*1658*	*140*	*55*	*172*	*260*

Bold numbers indicate the two highest percentages per column. n is: number of sequences used for the analysis.

**Table 2:** Relative distributions of sources in percentage within each assemblage.

Source	Cat	Cattle	Dog	Goat & Sheep	Human	Pig	Wildlife	Total (n)
A	***19***	***10***	*10*	***8***	***19***	***9***	***24***	1206
B	2	2	10	1	**62**	1	22	1037
C	8	0	**86**	0	0,3	0	5	200
D	5	0	**88**	0	0,5	2	5	224
E	0,4	***31***	0,4	***34***	0,1	***32***	3	722
F	**100**	0	0	0	0,4	0	0	80
G	0	0	0	0	0	0	100	28

The relative distributions are corrected the different numbers of isolates within each source.

Calculations are based on the percentages of Table 1, omitting “Other” as source. For example, The relative percentage of assemblage A found in cats is: 43/(43+23+23+17+43+21+70+54)*100. In non-human primates (Source: “Other”), assemblage B is the most prevalent *G. duodenalis* found. Bold numbers indicate the two highest percentages per column. n is: number of sequences used for the analysis.

The host distribution of assemblage A is less restricted than B, where companion animals (29%) livestock (27%) and wildlife (22%) have a comparable prevalence of assemblage A as in humans (19%). This result suggests that humans are the major source of assemblage B, but that domestic animals play a major role in the host range of assemblage A.

For those isolates which were characterized at two or more loci (n = 908), the assignment to a specific assemblage obtained at one locus was inconsistent with that obtained at another locus in 13% of them (Table 3). Similar results have been reported in previous studies, using the same markers as those in the present study [20],[25],[34]. This finding was particularly frequent in isolates from dogs (∼34%) where, depending on the markers used, isolates are typed as either host-adapted assemblages C and D, or as assemblage A and B (Table 4). Also in ∼12% of the human isolates (n = 392) mixing of assemblages was observed between A and B. As sexual recombination between different assemblages has not been unequivocally demonstrated [23],[35], these cases are more likely to represent mixed infections.

**Table 3:** Mixtures of assemblages in individual isolates with more than two markers.

Source	Cat	Cattle	Dog	Goat & Sheep	Human	Pig	Water	Wildlife	Other	TYptal
Mixed (n)	2	6	45	1	46	4	0	3	14	121
2^+^ Markers (n)	35	144	134	49	392	56	0	52	53	908
Mixed (%)	6%	4%	34%	2%	12%	7%	ND	6%	26%	100%

121 of the 908 isolates with two or more markers (13,3%) contain a mixture of two assemblages. In 3 isolates from dogs, mixtures of three assemblages were present in (ABC and BCD).

**Table 4:** Combination of mixed assemblages found in individual isolates.

	B	C	D	E	F
A	66	7	7	12	0
B	-	4	4	1	0
C	-	-	15	0	0
D	-	-	-	2	1

Only isolates with more than two markers and with inconsistent assemblage typing at different markers are used.

### Typing at the sub-assemblage level

Sub-groups within assemblages A and B were originally defined by isoenzyme analysis of laboratory-adapted strains, and classified into AI and AII, BIII and BIV [28]. Importantly, other subgroups were observed in a more recent study also based on isoenzyme analysis, and some appear to be host specific [15]. DNA sequence analysis of a smaller number of these isolates confirmed the existence of these subgroups in assemblage A and B at different loci [15]. More recently, a third sub-assemblage within assemblage A (referred to as AIII) was identified, and appears to be specifically associated with wild hoofed animals [22],[36],[37]. The SSU-rDNA locus showed too little variability among assemblage A and B isolates to perform analysis at the sub-assemblage level, whereas sufficient genetic variation was observed at the other three loci [22]. In companion animals and in livestock infected with assemblage A, approximately three quarter of the sequences corresponded to sub-assemblage AI, and the remaining quarter to sub-assemblage AII (Table 5). The opposite was found in human isolates: approximately one quarter of the sequences was identified as sub-assemblage AI and three quarter as sub-assemblage AII. The AIII sub-assemblage was mostly found in wildlife, a few cows and in a single cat isolate, but never in humans. In human isolates with assemblage B, sub-assemblage BIII and BIV were found with a very similar frequency (Table 6). In some wild animals (beaver, muskrat), sub-assemblage BIV was predominantly found. Monkeys and marine animals [22],[38],[39],[40],[41], which together represent the majority of the category “others”, were both infected with sub-assemblage BIII and BIV. Thus, at this level of resolution, *G. duodenalis* sub-assemblage AI, AII, BIII and BIV are potentially zoonotic, whereas sub-assemblage AIII is found exclusively in animals.

**Table 5:** Distribution of sub-assemblages AI, AII, and AIII in different sources.

Source	Cat	Cattle	Dog	Goat, Sheep	Human	Pig	Wildlife	Other
AI	**69%**	**62**%	**73%**	**78**%	25%	**86**%	**44**%	**55**%
AII	25%	35%	27%	22%	**75**%	14%	3%	**45**%
AIII	5%	4%	0%	0%	0%	0%	**52**%	0%
Total (n)	59	113	120	36	594	14	86	80

Sequences of BG (n = 493), GDH (n = 322) and TPI (n = 308), belonging to assemblage A, were subdivided into sub-assemblages AI, AII, and AIII based on SNPs [22]. Distribution of sub-assemblages within a source is calculated as their percentage of occurrence in the three cumulative markers. Bold numbers indicate the (two) highest percentage(s) per column.

**Table 6:** Distribution of sub-assemblages BIII and BIV in different sources.

Source	Dog	Human	Wildlife	Other
BIII	27%	**56%**	6%	43%
BIV	**73%**	44%	**94%**	**57%**
Total (n)	51	787	31	151

Sequences of BG (n = 254), GDH (n = 366) and TPI (n = 412), belonging to assemblage B, were subdivided into sub-assemblages BIII and BIV based on SNPs [22]. Distribution of sub-assemblages within a source is calculated as their percentage of occurrence in the three cumulative markers. Bold numbers indicate the highest percentage per column.

The geographic distribution of sub-assemblages AI and AII in humans and companion animals/livestock was compared. In companion animals/livestock infected with assemblage A, the majority was sub-assemblage AI, and the minority was sub-assemblage AII (Table 7). This distribution was found globally, suggesting that sub-assemblage AI has a preference for companion animals/livestock. Except for Asia and Australia, the opposite was found in humans: the majority was sub-assemblage AII, and the minority was sub-assemblage AI. These data show that the three *G. duodenalis* sub-assemblages A predominantly/preferentially cycle within defined hosts (AI in livestock, AII in humans, AIII in wildlife), and that these cycles do not interact significantly. The geographic distribution of sub-assemblages BIII and BIV in humans showed marked differences between continents. In Africa, infection with *G. duodenalis* assemblage B, sub-assemblage BIII is more prevalent (81%) than infection with sub-assemblage BIV (19%), whereas the opposite is found in North-America where 86% of infections are associated with sub-assemblage BIV, and only 14% with sub-assemblage BIII (Table 8). A more balanced distribution is found in Europe and Australia.

**Table 7:** Geographic distribution of AI and AII in humans and domestic animals.

Human	Africa	Asia	Australia	Europe	Middle east	C/S-America	N-America
AI	12%	**60%**	**69**%	14%	13%	42%	44%
AII	**88%**	40%	31%	**86**%	**88%**	**58%**	**56%**
Total (n)	73	5	26	295	16	160	16
Domestic animals	Africa	Asia	Australia	Europe	Middle east	C/S-America	N-America
AI	**67%**	**100%**	**92%**	**67%**	0	**77%**	**65%**
AII	33%	0%	8%	33%	0	23%	35%
Total (n)	3	9	12	334	0	30	84

Data from Table 5 were grouped in “humans” and “domestic animals”, the latter represents cats, cattle, dogs, goats and sheep, and pigs. Distribution of sub-assemblages within a geographic region is calculated as their percentage of occurrence in the three cumulative markers. Bold numbers indicate the (two) highest percentage(s) per column.

**Table 8:** Geographic distribution of BIII and BIV in humans.

Human	Africa	Asia	Australia	Europe	Middle east	C/S-America	N-America
BIII	**81%**	**68%**	**52%**	**49%**	**63%**	**79%**	14%
BIV	19%	32%	**48%**	**51%**	37%	21%	**86%**
Total (n)	54	47	31	508	8	124	14

Distribution of sub-assemblages in humans within a geographic region is calculated as their percentage of occurrence in the three cumulative markers. Bold numbers indicate the (two) highest percentage(s) per column.

The finding of a mixture of assemblages in a significant fraction of individual isolates prompted us to investigate whether this occurred at the level of sub-assemblages. In isolates analysed at two or more loci, sub-assemblage results obtained at the different loci were compared. Mixtures were found between AI and AII, and between AI and AIII. No mixtures were detected between AII and AIII. Within assemblage A, 5.4% of mixtures were observed between sub-assemblages AI and AII. Remarkably, mixtures between BIII and BIV characterized 30.3% of the isolates. Analysis of human isolates showed that an infection with AI alone occurs as often as an infection with a mixture of AI and AII (Table 9). A similar situation occurred with sub-assemblage BIII and BIV: an infection with BIV occurs as often as an infection with a mixture of BIII and BIV.

**Table 9:** Mixing of A and B sub-assemblages within isolates.

All isolates				
Assemblage A	AI	AII	AIII	
AI	102	19	3	
AII		231	0	
AIII			38	
**Assemblage B**	**BIII**	**BIV**		
BIII	199	144		
BIV		132		
**Human only**	**AI**	**AII**	**BIII**	**BIV**
AI	12	12	5	2
AII		226	29	8
BIII			193	107
BIV				105

Mixing within individual isolates typed at two or more markers was investigated by comparison of the sub-assemblage assignment of individual markers within one isolate. Mixing between markers is shown in bold. In total 5.4% of mixing was observed between sub-assemblage AI and AII (n = 352 sequences). Mixing was detected between AI-AII, and AI and AIII, but not between AII and AIII. Mixing between sub-assemblage BIII and BIV was found in 30.3% of isolates (n = 475 sequences). In human isolates the mixing between all sub-assemblages within isolates was determined.

### Single versus multi-locus typing at the isolate level

Sequence heterogeneity was also observed within each sub-assemblage, and those genetic variants are referred here as subtypes. In order to determine the zoonotic potential at this level, subtypes were assigned to groups of sequences, on the basis of similarity [22],[30],[31]. Thus, sequences that differ for a single nucleotide difference defined two subtypes. For example, at the SSU-rDNA locus, 15 subtypes were found among assemblage A isolates (Table 10). Of these, 3 and 7 subtypes were exclusively found in humans or in animals, respectively, whereas 5 subtypes contained both human and animal isolates. Notably, these 5 subtypes correspond to 92% of the isolates (humans and animals). Genetic variability at each of the other three loci defined several subtypes (between 3 and 18) in both assemblages A and B, and, as subtypes comprises both human and animal isolates, it is possible to infer a zoonotic potential. Subtypes were also determined for assemblages C to F. The subtypes of assemblage C, D and E found in a few human isolates did not match any of the subtypes found in animals. However, several subtypes of assemblage F found in humans at the BG locus were identical to subtypes found in cats [32].

**Table 10:** Potential zoonotic subtypes using one, two or three markers.

Subtype (isolates)	Assemblage	Human	Animal	H & A	Total
SSU-rDNA	A	3 (2%)	7 (5%)	**5 (92%)**	15 (n = 133)
	B	9 (17%)	3 (2%)	**3 (80%)**	15 (n = 133)
BG	A	29 (16%)	39 (15%)	**12 (69%)**	80 (n = 488)
	B	45 (40%)	8 (5%)	**10 (55%)**	63 (n = 211)
GDH	A	9 (15%)	24 (23%)	**7 (62%)**	40 (n = 331)
	B	68 (58%)	18 (13%)	**14 (29%)**	100 (n = 252)
TPI	A	12 (11%)	25 (19%)	**5 (70%)**	42 (n = 266)
	B	66 (29%)	34 (14%)	**18 (57%)**	118 (n = 344)
rDNA-BG	A	6 (76%)	4 (15%)	**1 (9%)**	11 (n = 33)
	B	15 (83%)	4 (11%)	**1 (7%)**	20 (n = 46)
rDNA-GDH	A	17 (58%)	7 (32%)	**2 (11%)**	26 (n = 57)
	B	30 (92%)	3 (5%)	**1 (3%)**	34 (n = 63)
rDNA-TPI	A	6 (73%)	5 (15%)	**1 (12%)**	12 (n = 33)
	B	16 (38%)	6 (13%)	**2 (50%)**	24 (n = 48)
BG-GDH	A	22 (51%)	18 (34%)	**2 (15%)**	42 (n = 137)
	B	48 (84%)	10 (16%)	0	58 (n = 95)
BG-TPI	A	17 (49%)	10 (25%)	**3 (26%)**	30 (n = 124)
	B	40 (75%)	10 (23%)	**1 (2%)**	51 (n = 83)
GDH-TPI	A	16 (49%)	12 (29%)	**2 (22%)**	30 (n = 113)
	B	38 (82%)	12 (18%)	0	50 (n = 88)
rDNA-BG-GDH	A	15 (78%)	5 (22%)	0	20 (n = 27)
	B	21 (94%)	2 (6%)	0	23 (n = 34)
rDNA-BG-TPI	A	10 (81%)	3 (19%)	0	13 (n = 21)
	B	13 (77%)	6 (23%)	0	19 (n = 27)
rDNA-GDH-TPI	A	12 (83%)	4 (17%)	0	16 (n = 25)
	B	16 (94%)	2 (6%)	0	18 (n = 32)
BG-GDH-TPI	A	23 (62%)	10 (38%)	**2 (15%)**	35 (n = 101)
	B	31 (78%)	8 (22%)	0	39 (n = 56)

A subtype is a group of sequences (isolates) which are similar. Subtypes of assemblages A and B were identified using a similarity matrix of individual loci. The similarity matrix was calculated using UPGMA as a first criterion, and “most identical matches” as secondary criterion (see Methods). Subtypes with two or three loci were identified by combining the subtyping results of the individual markers. The column Human contains the number of subtypes which members were only human isolates. The column Animal contains the number subtypes, which members were only of animal origin. The column H & A contains the number of subtypes, which consist of both human and animal isolates. Total displays the total number of isolates per (combination of) markers. Between brackets is the percentage (%) or the total number (n) of isolates, which correspond to the number of subtypes. Subtypes of isolates with more than one marker were subsequently assigned by combining the subtypes of each marker. rDNA stands for SSU-rDNA.

In order to increase the accuracy of genotyping of isolates at this level, subtypes from two or three loci were combined to define multi-locus genotypes (MLGs). 41 sequences, which could not be unequivocally assigned at the level of assemblage, were excluded from the analysis. Combining SSU-rDNA and BG was possible for 33 isolates of assemblage A, and defined 11 MLGs (Table 10). With this combination only one MLG of assemblage A was potentially zoonotic. The combination of SSU-rDNA and BG for assemblage B also generated a single potentially zoonotic MLG out of 20 MLGs. This MLG was found in 3 out of 46 isolates of assemblage B. The same approach was used for all possible combinations of the 4 markers (Table 10). When using two markers, the number of potentially zoonotic subtypes and the percentage of corresponding isolates decreased significantly. Still, potential zoonotic subtypes of both assemblage A and B were found when using two markers. When subtypes from three loci are combined, two MLGs of assemblage A are potentially zoonotic, and none of assemblage B. These cases have been described before. In Italy, an isolate from a cat (ISSGdA107) has a MLG belonging to sub-assemblage AII [22]. Human isolates from Belgium, Germany, The Netherlands, Italy, France, Nicaragua, and Australia, have the same MLG. The other case is based on two axenic strains that have a MLG belonging to sub-assemblage AI These two isolates, Portland and Ad-1, were originally isolated from human patients in the USA and Australia, respectively [15]. Remarkably, the animal (mostly cattle) isolates having this MLG are from Canada, Italy and Sweden.

There are several technical explanations for the relatively low number of zoonotic MLGs as defined using three loci. Most importantly, the number of isolates typed at this level is still relatively small compared to the number of subtypes defined. Furthermore, most MLGs are from human isolates, particularly for assemblage B. Indeed, for many animal isolates of assemblage A or B, only one or two markers were sequenced, and, in some cases, the mixture of zoonotic and non-zoonotic assemblages prevents an unambiguous identification of the MLGs. An alternative, but less accurate, approach for the identification of potential zoonotic MLGs is to combine the zoonotic information of subtypes of individual markers (Table 10, row 1–4). Isolates with 3 markers (BG, GDH and TPI) were considered as potentially zoonotic when all three markers were found to be zoonotic individually. For assemblage A, 36% (n = 101) of isolates with 3 markers was found to be zoonotic. For assemblage B, 4% (n = 56) was potentially zoonotic (Table 11).

**Table 11:** Number of potential zoonotic isolates with 3 markers.

Assemblage A	Human	Animal	H & A	Unassigned
Human	0	0	**26**	39
Cat	0	2	**1**	4
Cattle	0	0	**4**	0
Goat &sheep	0	0	**2**	5
Wildlife	0	11	**2**	1
Other	0	1	**1**	2
Total (Isolates)	0	14	**36**	51
**Assemblage B**	**Human**	**Animal**	**H & A**	**Unassigned**
Human	3	0	**1**	39
Wildlife	0	0	**0**	1
Other	0	1	**1**	10
Total (Isolates)	3	1	**2**	50

(n = 101) for assemblage A, and 4% (n = 56) for assemblage B, were potentially zoonotic. The zoonotic information of subtypes from each marker (see Table 10) was used to determine the zoonotic potential of isolates with 3 markers (BG, GDH and TPI). Isolates were considered zoonotic (H & A) when the subtypes of all the 3 markers were designated individually as potentially zoonotic. The column Human and Animal contains the number of isolates which subtypes were exclusively found in humans or animals, respectively. The column Unassigned contains the number of isolates from which the subtypes of one or two markers was zoonotic and the other(s) specifically for humans or animals. Thus, 36%.

### Sequences containing ambiguous nucleotides

The presence of heterogeneous sequencing profiles (characterized by two overlapping nucleotide peaks at specific positions) has been reported in several papers from different research groups [19],[22],[32],[42]. Besides the quality of the sequencing reaction itself, two explanations can be given for the presence of those mixed profiles: allelic sequence heterozygosity (ASH) and mixed infections. *Giardia* has two diploid nuclei, which may accumulate specific mutations independently, and this generates ASH [23]. The fact that *G. duodenalis* isolates display a very low level of ASH, initially based on the analysis of few isolates and genetic loci [35],[43], has been confirmed by the analysis of the complete WB genome, a strain belonging to assemblage A, sub-assemblage AI [43]. Albeit limited by the small number of loci, and by the difficulty in distinguishing ASH from mixed infections, the data presented in Table 12 clearly shows that heterogeneous sequencing profiles occur much more often in isolates of assemblages B, C, and D than in those from assemblage A, E and F. The number of heterogeneous positions also varied among the loci analysed and the positions involved often coincide with polymorphic sites among different subtypes.

**Table 12:** Sequences containing ambiguous nucleotides.

	SSU-rDNA	BG	GDH	TPI	Total (%)
A	13% (165)	6% (516)	2% (338)	4% (271)	**5% (1290)**
B	16% (161)	16% (247)	32% (345)	16% (398)	**21% (1151)**
C	5% (65)	24% (42)	15% (53)	47% (45)	**20% (205)**
D	0% (39)	31% (81)	15% (89)	42% (19)	**20% (228)**
E	0% (200)	11% (205)	29% (237)	7% (95)	**10% (737)**
F	0% (13)	8% (24)	6% (36)	8% (13)	**6% (86)**

Occurrence of heterogeneous positions in the sequences of beta-giardin (BG), glutamate dehydrogenase (GDH) and triose phosphate isomerase (TPI) genes as found in isolates of assemblages A to F.

Data were taken from the ZoopNet database (February 2009). Between brackets is the total number of sequences used to calculate the percentage of sequences with heterogeneous positions.

The occurrence of ASH complicates the assignment of isolates to specific subtypes, especially for assemblage B. Therefore, the occurrence of zoonotic subtypes within assemblage B was tested after the exclusion of ambiguous nucleotides. The BG, GDH, and TPI sequences from a total of 117 assemblage B isolates (100 from humans, and 17 from animals) were merged and clustered. No zoonotic subtypes were detected. When all isolates (n = 199) typed with 2 markers (BG-GDH, BG-TPI, or GDH-TPI) were included in the analysis, 7% were compatible with zoonotic potential. Interestingly, these isolates were from zoo animals and a rabbit.

### Genetic heterogeneity

A measure of the genetic diversity of a locus can be estimated by the number of subtypes corrected for the number of isolates. This was achieved by dividing the number of isolates without ambiguous nucleotides (Table 13) by the number of subtypes. The lowest genetic variability was found at the SSU-rDNA locus. Although 15 subtypes were identified at SSU-rDNA for both assemblage A and B, sequence variation, no distinction could be made between sub-assemblages. Most of the sequence variation found at SSU-rDNA was caused by a minority of the isolates. The genetic variability of the other 3 markers varied only a little from each other. Remarkably, the genetic variability at each marker in assemblage A subtypes was ∼2-fold lower than that found in assemblage B subtypes. The genetic distance within assemblage A was higher than within assemblage B (Table 13).

**Table 13:** Genetic heterogeneity of assemblage A and B.

Subtypes (isolates)	Assemblage	Diversity Isolates/subtypes	Similarity (%)
SSU-rDNA	A	8.9 (133/15)	98.2*
	B	8.9 (133/15)	98.5*
BG	A	6.1 (488/80)	98.1 (98.8)
	B	3.4 (211/63)	98.9
GDH	A	8.3 (331/40)	96.2 (98.7)
	B	2.5 (252/100)	96.6
TPI	A	6.3 (266/42)	97.0 (99.2)
	B	2.9 (344/118)	97.7

The genetic diversity was measured by dividing the total number of isolates by the total number of subtypes. High numbers represent low genetic diversity. Sequences with ambiguous nucleotides were not taken into account. Percentage of similarity is based on multiple alignment of UPGMA. Values in brackets are without AIII. *With SSU-rDNA no differences were observed between AI, AII and AIII, and between BIII and BIV.

### Phylogenetic analysis of assemblages A and B

**Figure 1:**
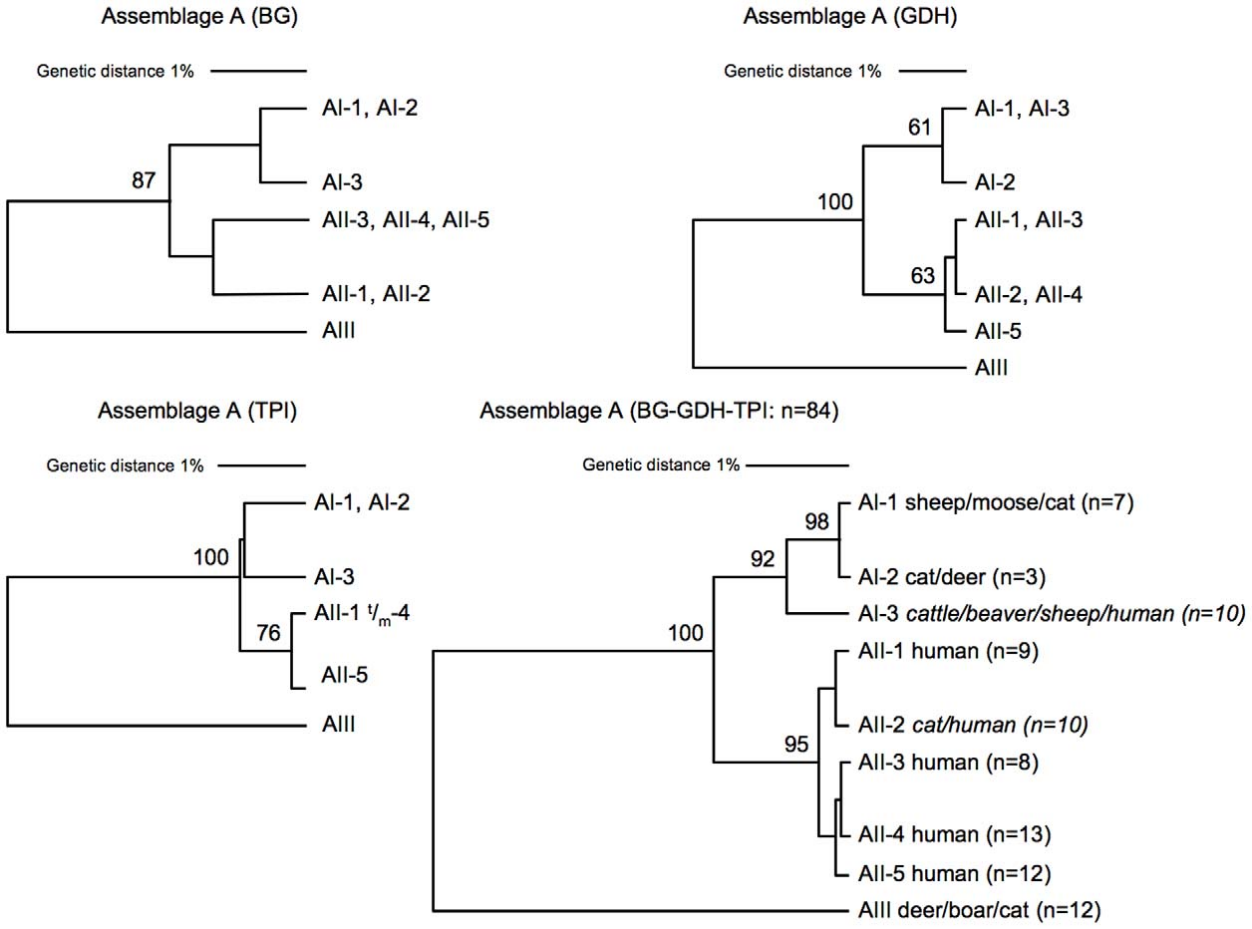
Phylogenetic analysis of assemblage A. Phylogenetic trees of 84 isolates with 3 markers were inferred using Unweighted Pair Group Method with Arithmetic mean, corrected by complete linkage, which uses the lowest similarities found between two clusters. Individual and merged BG, GDH and TPI nucleotide sequences were used. Bootstrap values were calculated by the analysis of 1000 replicates. Only bootstrap values >60 are shown. The phylogenetic analysis of assemblage A shows that the three sub-assemblages clustered together with high bootstrap support (i.e., they are monophyletic). The genetic diversity of the multi-locus genotypes (isolates/subtypes: 9,3) is relatively low (see Table 13), and the maximum genetic distance is 4,0%.

The multi-locus analysis of field isolates may not represent *G. duodenalis* genotypes as they could consist of a mixture of several *G. duodenalis* (sub)species. To identify multi-locus genotypes among isolates of assemblage A, the sequences of the BG, GDH, and TPI loci from isolates with matching assignment were merged, a multiple alignment was generated and trees were constructed using complete linkage. To increase the accuracy of the analysis, only multi-locus genotypes found in more than one isolate were selected. In total 9 MLGs were identified from 84 isolates for assemblage A (Figure 1). To evaluate the robustness of the inferred relationships within assemblage A, trees were also generated from each marker. The clustering generated from the individual markers was congruent with the clustering of multi-locus profile (Table 14). These analyses confirmed the existence of three monophyletic sub-assemblages at each marker. However, the sequence variation at each locus was too low to discriminate between the different subtypes *within* sub-assemblage AI and AII. For example, subtype AI-1 cannot be distinguished from AI-3 with GDH, and AI-1 is identical to AI-2 when using BG and TPI. Two genotypes were identified, AI-III, and AII-II, which contained both human and animals isolates, which is in agreement with the MLGs identified previously (Table 10).

**Table 14:** Congruence of phylogenetic analysis of assemblage A and B.

Ass A	BG	GDH	TPI	Merge
BG	100	86	96	96
GDH		100	90	97
TPI			100	97
Merge				100
Ass B	BG	GDH	TPI	Merge
BG	100	12	31	62
GDH		100	6	46
TPI			100	74
Merge				100

**Figure 2:**
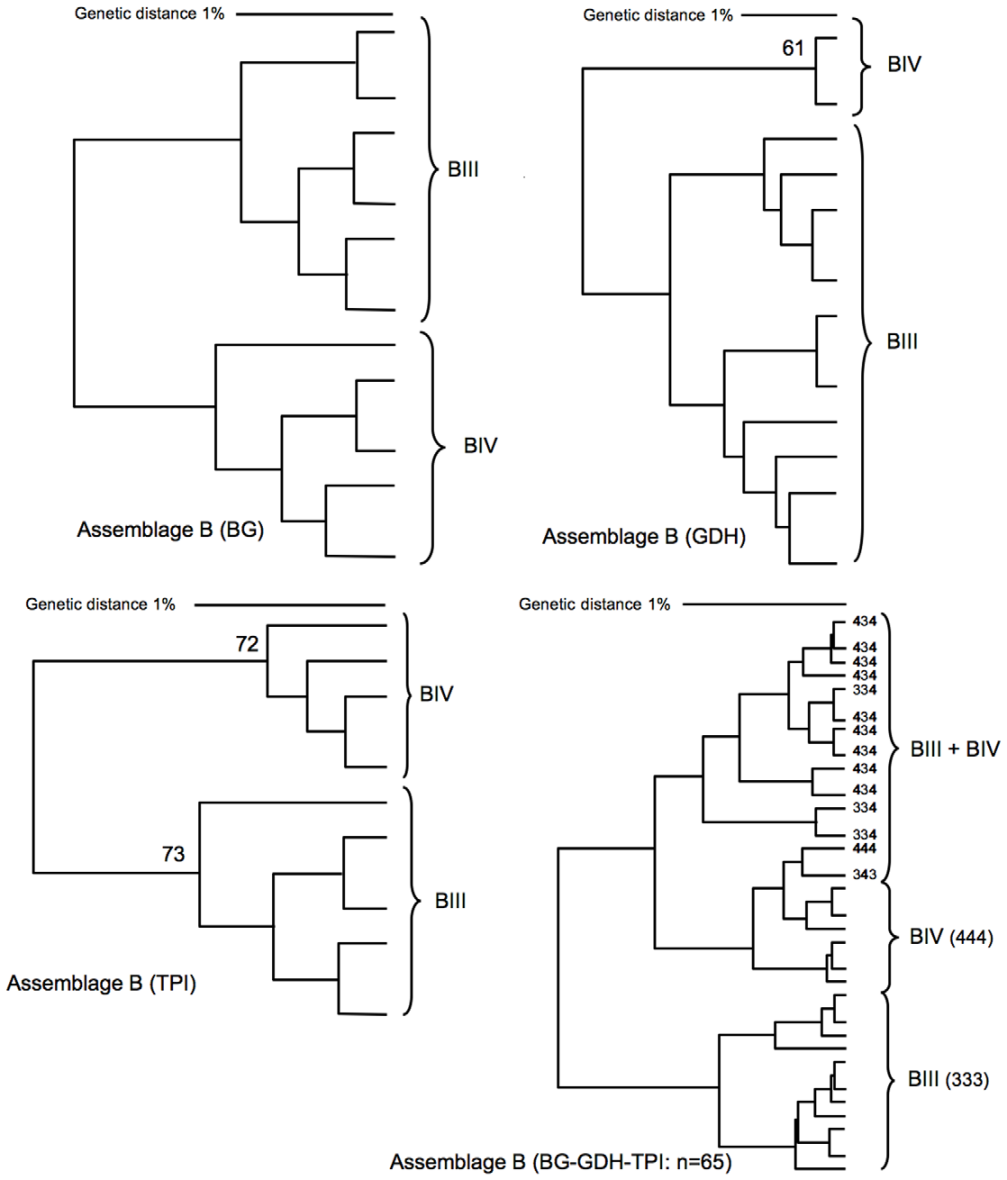
Phylogenetic analysis of assemblage B. Phylogenetic trees of 65 isolates of assemblage B with 3 markers were constructed using Unweighted Pair Group Method with Arithmetic mean, corrected by complete linkage, which uses the lowest similarities found between two clusters. Individual and merged BG, GDH and TPI nucleotide sequences were used. Bootstrap values were calculated by the analysis of 1000 replicates. Only bootstrap values >60 are shown. The phylogenetic analysis of the merged sequences shows significant mixtures of the BIII and BIV sub-assemblages. The genetic diversity of the multi-locus genotypes (isolates/subtypes: 2,1) is relatively high (see Table 13), and the maximum genetic distance is 1,7%.

Congruence is calculated from the cluster analysis of 3 markers (BG, GDH, TPI) and of their merge. See also Figure 1 (assemblage A) and Figure 2 (assemblage B).

A similar analysis was performed for assemblage B isolates. In total 31 genotypes were identified from 65 isolates (Figure 2). The clustering generated from individual markers was able to discriminate sub-assemblage BIII from BIV, but with low bootstrap values, especially for BG. However, multi-locus genotyping of assemblage B was inconsistent with genotyping at the sub-assemblage level: significant mixing (∼30%) of BIII and BIV was observed. In contrast to assemblage A, clustering from individual loci of assemblage B was incongruent with clustering of multiple loci (Table 14). These results are consistent with the multi-locus subtyping of isolates: In assemblage A, mixing is less frequently observed than in assemblage B (Table 9). Removal of the “mixed MLGs” from the genotyping analysis did not alter the outcome of the analysis significantly: The bootstrap values as well as the congruency remained low (not shown). Compared to assemblage A, the MLG diversity (number of genotypes) of assemblage B is 4 times higher, but their genetic distance is two times lower, both at the level of individual markers and at the level of MLG (Figures 1 and 2).

## Discussion

In the present study, the zoonotic potential, genetic diversity, and the geographic distribution of *G. duodenalis* genotypes from the ZOOPNET-database were assessed. Accurate molecular typing is imperative for unraveling the intricate epidemiology of giardiasis. Molecular markers should be able to discriminate between morphologically identical isolates that may differ for important properties, like virulence and host-specificity. The genes used in this study have housekeeping functions and are presumably not directly linked to virulence and host-specificity. The discriminatory properties of the commonly used diagnostic markers of *G. duodenalis* have not been investigated systematically. Here, different molecular typing methods were used to address the discriminatory properties of SSU-rDNA, BG, GDH and TPI. Typing at the level of assemblages is relatively straightforward, and can be achieved with all four markers. Importantly, assemblages C, D, E and F are found in rare human cases (0.8% of human cases). These findings demonstrate that *G. duodenalis* assemblages C to F can indeed infect humans. Since human infection with these assemblages occurs infrequently, it seems that the host-range of *G. duodenalis* may be determined by more factors than the host-parasite interaction alone. It is also unclear whether human infections with assemblages C to F result in disease. Typing at the level of sub-assemblages was only possible for BG, GDH and TPI, but not for SSU-rDNA, because the SSU-rDNA locus showed too little intra-assemblage variability in both assemblage A and B (see also [22]).

### Assemblage A

Significant differences were found between the sub-assemblages AI, AII and AIII. Although sub-assemblages AI and AII are found in both humans and animals, sub-assemblage AI is preferentially found in livestock and pets whereas sub-assemblage AII is predominantly found in humans. Sub-assemblage AIII is almost exclusively found in wild hoofed animals, and is most likely a host-adapted genotype. Several potential zoonotic subtypes, which correspond to the majority of the isolates, were identified at the level of individual markers (Table 10). However, combining the subtype information of the available markers of individual isolates (MLG) resulted in only two potentially zoonotic genotypes within assemblage A. Thus, the most important conclusion is that analysis of single markers is inaccurate for molecular epidemiological studies. This finding is consistent with the phylogenetic analysis of assemblage A: the genetic variation found in individual markers is too low to allow discrimination of different genotypes (Figure 1). Conversely, many subtypes for assemblage A were identified for each marker (Table 10: 15 for SSU-rDNA, 80 for BG, 40 for GDH, and 42 for TPI). Subtyping is based on similarity, and a single point mutation has been considered sufficient to describe a new subtype. For all markers it was found that only a minority of subtypes corresponded to the majority of isolates and that the majority of subtypes were found in only one or two isolates. Whether all these subtypes correspond to new genotypes or whether some of them will turn out to be (sequence) artifacts is unclear. The significance of all these subtypes will become clearer when more molecular epidemiological data are added to the database. From the six MLGs defined within assemblage A, two are potentially zoonotic. Genotype AI-3 consisted mostly of animal isolates and of a few human (axenic) isolates, whereas AII-2 consisted predominantly of human isolates and a single cat isolate. These findings are in agreement with the preferential distribution of AI and AII found at the level of sub-assemblages. Since the number of MLG isolates is relatively small, especially for pet isolates typed with three (consistent) markers, more genotypes with zoonotic potential may exist. The assumption is that genetically identical *G. duodenalis* found in both humans and animals, are zoonotic. Remarkably, the isolates having zoonotic potential were not epidemiologically linked (i.e. same location, same study). These findings highlight the global distribution of these *G. duodenalis* genotypes, but provide little evidence for zoonotic transmission.

### Assemblage B

The host distribution of assemblage B is predominantly human and to a much lesser extent wildlife and dog (Table 2). Assemblage B is also found regularly in (captive) non-human primates. They generally do not play significant roles in the life cycle of G. duodenalis, which involve humans. The abundance of assemblage B in (captive) non-human primates may be due exposure to human sources. Alternatively, assemblage B is well-adapted to infect primates. Genotyping of assemblage B was more problematic. The genetic diversity (number of subtypes) and the percentage of sequences with mixed templates (ambiguous nucleotides) were ∼2,5 and 4 times higher than for assemblage A, respectively. The mixing of the sub-assemblages BIII and BIV within isolates was ∼30%, which is 6 times more than the mixing observed between sub-assemblages AI and AII. Furthermore, the 119 field isolates of assemblage B with 3 markers (BG, GDH, TPI) consisted of 102 humans, 13 primates, 2 zoo animals, one guinea pig and one rabbit. Relevant animal sources, in particular dogs and marine animals [38] are present in the database, but are not typed with the 3 markers of assemblage B. Together, these factors hamper the precise assignment of isolates at the assemblage or subtype level. Typing with two but not with three markers resulted in the identification of a few potentially zoonotic MLGs. Alternative approaches, e.g. removal of ambiguous nucleotides or estimation of potential zoonotic MLGs by combining the zoonotic information from individual markers, resulted in the identification of potential zoonotic genotypes, which corresponded to only 4–7% of the isolates of assemblage B. All in all, no clear genotypes could be inferred for assemblage B, and no distinction between zoonotic and host-adapted genotypes could be made within assemblage B.

### Mixed infections and allelic sequence heterozygosity

Two principal mechanisms can explain the occurrence of ambiguous nucleotides and the inconsistent assignment of single isolates at the level of both assemblage and sub-assemblage: (i) “true” mixed infections; and ii) allelic sequence heterozygosity (ASH). The presence of more than one *G. duodenalis* type during a symptomatic infection has important implications for the etiology of giardiasis: it is unclear how humans and animals become infected with two or more *G. duodenalis* types. Subjects may be infected simultaneously with different *Giardia* assemblages (or even subtypes), because of environmental mixing, for example in water. Alternatively, subjects are asymptomatically infected with one *Giardia* assemblage, but become ill/symptomatic from a second infection with another *Giardia* assemblage. The latter hypothesis is supported by the finding of asymptomatic subjects [6],[7],[12],[14]. The occurrence of mixed infections has important epidemiological implications. Using only one marker for the assignment of isolates to specific (sub)-assemblages is not always reliable, as different markers can give different results. For example, isolates can be typed as “potentially zoonotic” with one marker, but as “host-adapted” with another. More reliable results are obtained when multiple markers are used for typing. On the other hand, “true” *G. duodenalis* genotypes are difficult to identify in mixed infections.

Allelic sequence heterozygosity (ASH) is not unusual for diplomonads, which have two diploid nuclei, and replicate asexually [1]. Indeed, in asexual eukaryotes, the two allelic gene copies at a locus are expected to become highly divergent as a result of the independent accumulation of mutations in the absence of segregation (Meselson's effect). Therefore, substantial genetic differences are expected to accumulate among the chromosome homologues in asexual organisms with a ploidy of two or higher [44]. However, the ASH found in the genome of *G. duodenalis* assemblage A is extremely low [43], but the mechanism(s) responsible remained undetermined. Based on the presence of ambiguous nucleotides in sequences derived from PCR products, it is to be expected that the ASH is higher in assemblages B, C, and D than in assemblages A, E and F (Table 11). Recent studies have shown that *G.duodenalis* may be able to undergo sexual reproduction, a phenomenon that can influence ASH levels [35],[45]. However, the frequency of recombination is not known, nor its impact on the etiology and epidemiology of giardiasis [11],[23].

### Future directions

The ZOOPNET-database is the largest molecular epidemiological database of *G. duodenalis* to date. Still, the limitations of this unique database are apparent. Currently, the database contains a heterogeneous geographic- and incomplete source distribution of a “limited” set of isolates. Furthermore, each isolate is characterized by a small set of epidemiological data and limited sequence data. Our aim is to expand and improve the ZOOPNET database: since the content of the ZOOPNET database is accessible via internet, scientists can use these data for their own epidemiological studies. The web-based ZOOPNET-database will remain accessible, and its interface will be soon improved. Both veterinary and public health researchers are welcome to submit their molecular epidemiological data on *G. duodenalis* and *Cryptosporidium* to ZOOPNET. The web-based ZOOPNET-database has a flexible content and provides a powerful tool for new (inter)national studies on giardiasis (and cryptosporidiosis).

## Supporting Information

Text S1 Contents of the *Giardia* database, geographical distribution of the *Giardia* isolates present in the database, and GenBank accession numbers of reference sequences.
(0.07 MB DOC)
