# Supplementary material for: Identification of Zoonotic Genotypes of Giardia duodenalis
Source: PLoS Negl Trop Dis. 2009 Dec 1;3(12):e558. doi: 10.1371/journal.pntd.0000558 (PMC2777335; doi:10.1371/journal.pntd.0000558)
Supplement: Text S1 — Contents of the Giardia database, geographical distribution of the Giardia isolates present in the database, and GenBank accession numbers of reference sequences. (0.07 MB DOC) [file pntd.0000558.s001.doc]

**Supporting Information for “Identification of zoonotic genotypes of *Giardia duodenalis”***

**Supplement Table I: Contents of the *Giardia* database (February 2009)** Number of sequences per isolate. Sequences of the 4 markers are included in the sequences of 3, 2 and 1 marker etc. Category “Other” includes for example zoo and marine animals.

| ***Sequences***  ***(per marker)*** | | Cat | Cattle | Dog | Goat &  Sheep | Human | Pig | Water | Wildlife | Other | Total |
| --- | --- | --- | --- | --- | --- | --- | --- | --- | --- | --- | --- |
| 1 | SSUrRNA | 21 | 155 | 112 | 8 | 247 | 71 | 18 | 39 | 22 | 693 |
|  | BG | 49 | 185 | 170 | 107 | 516 | 5 | 14 | 53 | 52 | 1151 |
|  | GDH | 60 | 182 | 172 | 34 | 496 | 62 | 2 | 51 | 86 | 1145 |
|  | TPI | 28 | 48 | 154 | 58 | 412 | 2 | 24 | 60 | 111 | 897 |
| 2 | BG-GDH | 24 | 38 | 68 | 23 | 246 | 2 | 0 | 37 | 28 | 466 |
|  | BG-TPI | 19 | 24 | 55 | 40 | 228 | 2 | 0 | 27 | 28 | 423 |
|  | GDH-TPI | 19 | 25 | 51 | 22 | 230 | 1 | 0 | 25 | 45 | 434 |
| 3 | BG-GDH-TPI | 18 | 23 | 39 | 21 | 179 | 1 | 0 | 24 | 25 | 330 |
| 4 | All 4 markers | 0 | 0 | 6 | 0 | 41 | 1 | 0 | 2 | 6 | 56 |
| ***All isolates*** | | ***101*** | ***403*** | ***414*** | ***137*** | ***978*** | ***81*** | ***58*** | ***122*** | ***182*** | ***2476*** |

**Supplement Table II: Geographical distribution of the *Giardia* isolates present in the database (February 2009)** Category “Other” includes for example zoo and marine animals.

| ***Geographicall regions*** | Cat | Cattle | Dog | Goat &  Sheep | Human | Pig | Water | Wildlife | Other |
| --- | --- | --- | --- | --- | --- | --- | --- | --- | --- |
| Africa | 1 | 0 | 1 | 0 | 93 | 0 | 4 | 0 | 0 |
| Asia | 1 | 27 | 12 | 0 | 42 | 0 | 10 | 3 | 1 |
| Australia | 4 | 24 | 7 | 0 | 47 | 0 | 0 | 8 | 2 |
| Europe-central | 18 | 1 | 60 | 0 | 76 | 2 | 2 | 4 | 3 |
| Europe-north | 18 | 172 | 38 | 26 | 127 | 75 | 0 | 21 | 8 |
| Europe-south | 9 | 47 | 122 | 58 | 115 | 2 | 20 | 21 | 41 |
| Europe-west | 15 | 94 | 122 | 38 | 240 | 2 | 0 | 16 | 10 |
| Middle-east | 0 | 0 | 0 | 0 | 24 | 0 | 0 | 0 | 0 |
| Middle/South America | 22 | 5 | 42 | 14 | 190 | 0 | 7 | 0 | 9 |
| North-America | 11 | 33 | 8 | 0 | 20 | 0 | 15 | 44 | 98 |
| Unknown | 2 | 0 | 2 | 1 | 4 | 0 | 0 | 5 | 10 |
| Total | **101** | **403** | **414** | **137** | **978** | **81** | **58** | **122** | **182** |

**Supplement Table II**I: Genbank accession numbers of reference sequences

| (Sub)assemblage | SSU-rDNA | BG | GDH | TPI |
| --- | --- | --- | --- | --- |
| AI  AII  AIII  BIII  BIV  C  D  E  F  G | M54878  AF199446  DQ100287  AF199447  AF113898  AF199449  AF199443  AF199448  AF199444  AF199450 | X85958  AY072723  DQ100288  AY072726  AY072725  AY545646  AY545647  AY072729  AY647264  EU769221 | M84604  AY178737  EU637582 AF069059  AY178738  U60983  U60986  AY178741  AF069057  AY178746 | L02120  U57897  DQ650648  AF069561  AF069560  AY228641  DQ246216  AY228645  AF069558  EU781013 |
